# Dynamic Remodeling of the Human Milk Serum Proteome Across Lactation: A Paired Two-Stage DIA Proteomic Study in Term and Preterm Mothers

**DOI:** 10.3390/nu18132199

**Published:** 2026-07-07

**Authors:** Nina Mól, Magdalena Zasada, Maciej Suski, Wojciech Zasada, Przemko Kwinta

**Affiliations:** 1 Department of Pediatrics, Jagiellonian University Medical College, Wielicka 265 Street, 30-663 Krakow, Poland; nina.mol@uj.edu.pl (N.M.); magdalena.zasada@uj.edu.pl (M.Z.); 2 Department of Pharmacology, Jagiellonian University Medical College, Grzegórzecka 16 Street, 31-531 Krakow, Poland; maciej.suski@uj.edu.pl; 3 Proteomics Laboratory, Centre for the Development of Therapies for Civilization and Age-Related Diseases CDT-CARD, Jagiellonian University Medical College, Skawińska 8 Street, 31-066 Krakow, Poland; 4 Clinical Department of Cardiology and Cardiovascular Interventions, University Hospital, 30-688 Krakow, Poland; zasada.wojciech@gmail.com; 5 KCRI, 30-347 Krakow, Poland

**Keywords:** human milk, proteomics, lactation, preterm birth, data-independent acquisition, mass spectrometry

## Abstract

Objectives: Human milk composition changes across lactation, but paired within-subject proteomic analyses comparing longitudinal trajectories in term and preterm milk remain limited. We aimed to characterize stage-associated proteomic changes within each cohort and determine whether longitudinal remodeling is shared or divergent between term and preterm lactation. Methods: In this single-center prospective study conducted at the Neonatal Intensive Care Unit, Jagiellonian University Medical College, Kraków, Poland (October 2020–November 2021), 40 lactating mothers (20 preterm, <32 weeks’ gestation, mean age 29.4 ± 6.1 years; 20 term, 37–42 weeks, mean age 30.2 ± 5.5 years) provided paired milk samples at ≤10 days postpartum and week 5. Milk serum proteomes were analyzed by quantitative data-independent acquisition mass spectrometry; differential abundance was assessed using two-sample *t*-tests with Storey false discovery rate correction (q < 0.05) and fold-change >1.5, followed by ClueGO pathway enrichment. Results: Stage-associated differential abundance was identified for 108 proteins in term milk (58 increased, 50 decreased) and 103 in preterm milk (64 increased, 39 decreased). Of these, 87 were shared between cohorts (80.6% of term, 84.5% of preterm set) with concordant directionality. Shared upregulated pathways included oxidative stress response and glycolysis (e.g., PRDX5, fold change 2.49, q = 0.044); shared downregulated pathways related to mucosal immunity (e.g., tenascin, fold change 9.61–11.41, q < 0.0001). Cohort-specific pathway signals were limited relative to shared remodeling. Conclusions: The human milk serum proteome undergoes substantial longitudinal remodeling in both term and preterm lactation, with most changes following a common temporal pattern; prematurity-related differences appear selective rather than global. These findings support lactation stage as a key determinant of milk proteomic composition and underscore the value of longitudinal, stage-aware study designs, although formal time-by-group interaction testing was not performed.

## 1. Introduction

The mammary gland is a dynamic, hormonally regulated organ whose structural and functional maturation underlies the capacity for milk synthesis and secretion. Reproductive and metabolic hormones, principally estrogen and progesterone during mammary development, and prolactin and oxytocin during established lactation, coordinate the proliferation, differentiation, and secretory activation of mammary epithelial cells [1,2]. Following parturition, withdrawal of progesterone permits prolactin-driven secretory activation, while infant suckling sustains milk synthesis and ejection through neuroendocrine reflexes; after weaning, declining prolactin secretion and local cytokine signaling trigger glandular involution [2]. This tightly regulated physiological process underlies not only the volume of milk produced but also the dynamic remodeling of its molecular composition throughout lactation.

The composition of human milk is, in turn, shaped by a complex interplay of maternal, infant, and methodological factors. Maternal characteristics such as gestational age at delivery, parity, body mass index, and diet, together with infant-related factors including birth weight and postnatal age, have all been associated with variation in milk macronutrient, micronutrient, and bioactive protein content, although the strength of evidence differs considerably across these factors [3]. Among these determinants, gestational age and stage of lactation are the two most consistently and robustly associated with compositional change, and are the principal axes examined in the present study.

Human milk itself is a dynamic and biologically complex fluid whose composition evolves throughout lactation. Beyond macronutrients, it contains a broad range of bioactive components, including proteins, human milk oligosaccharides, lipids, vitamins, minerals, immune and epithelial cells, extracellular vesicles, and numerous signaling molecules that collectively contribute to infant nutrition, immune development, and host defense. Human milk oligosaccharides alone account for up to 80% of the solid, non-lipid components of milk and act as prebiotics with immunoregulatory and antiallergic properties [4]. Vitamins and minerals, including vitamin A, vitamin D, zinc, and iron, support both basic and pathogen-specific immune function in the infant [5]. Milk also contains a heterogeneous population of epithelial, immune, and stem cells, together with extracellular vesicles, which mediate intercellular communication and deliver microRNA and protein cargo to the infant gut [6,7]. These cellular and bioactive components contribute to early-life immune programming, including epigenetic modulation of immune-related gene expression [8]. Among these components, milk proteins play critical roles in immune regulation, oxidative stress responses, tissue remodeling, and metabolic processes, and represent a major focus of proteomic investigations. Representative examples include secretory IgA, lactoferrin, and lysozyme, which contribute to immune regulation and antimicrobial defense [9]. Growth factors such as epidermal growth factor (EGF) and transforming growth factor-β (TGF-β) further support tissue maturation [10].

These protein components change substantially between early and later lactation stages. Proteomic and systematic analyses have demonstrated marked differences in milk protein composition across defined lactation windows, with substantial remodeling observed between early and mature milk [11,12,13]. For instance, concentrations of secretory IgA, IgM, and IgG decline sharply during the first two weeks postpartum, together with reductions in α-lactalbumin, lactoferrin, and β-casein, whereas lysozyme increases over the same period [11]; similarly, lactoferrin, immunoglobulins, and clusterin reach their highest concentrations in colostrum before declining in transitional and mature milk [13]. Longitudinal investigations indicate that bioactive and immune-related proteins undergo coordinated quantitative modulation throughout early and mid-lactation [11].

Gestational age introduces a further layer of variability. Comparative studies of preterm and term milk have reported variations in immune-related and structural proteins: colostrum from mothers of preterm infants shows higher concentrations of IL-6, TGF-β1, and TGF-β2 than term colostrum, whereas IgA, IL-8, IL-10, and TNF-α are reduced in very preterm colostrum relative to term colostrum [14], and proteomic profiling has identified differential expression of proteins primarily involved in immune response and metabolic processes between term and preterm milk [15]. Both gestational age and lactation stage thus contribute independently to compositional variability, including macronutrient content [16].

Recent cohort-based initiatives emphasize the importance of studying human milk as an integrated biological system using structured longitudinal designs to disentangle temporal and interindividual variability [17]. Despite increasing use of high-resolution mass spectrometry, paired within-subject proteomic analyses across predefined lactation stages in both term and preterm cohorts remain limited. In a previous stage-stratified analysis of this cohort, we assessed proteomic differences between preterm and term milk at two predefined lactation stages. Between-group differences were modest and varied across time points, underscoring the importance of stage-specific interpretation. However, cross-sectional analyses do not clarify whether temporal changes occur similarly within each cohort. A substantial proportion of these changes may reflect a conserved temporal remodeling program in the human milk proteome. This study extends previous proteomic investigations by applying paired within-subject longitudinal proteomic analysis of term and preterm human milk using high-resolution quantitative data-independent acquisition mass spectrometry. In contrast to previous studies based on cross-sectional or pooled-sample designs, this approach allows temporal proteomic remodeling to be distinguished from interindividual and between-group variability at the level of the individual mother. By analyzing parallel cohorts under identical analytical conditions, the study further enables a direct assessment of whether longitudinal remodeling is shared or cohort-specific—a question that cross-sectional designs cannot address. These features provide methodological advantages compared with previous cross-sectional analyses and facilitate a more direct assessment of longitudinal proteomic remodeling. The findings have potential relevance for the development of stage-aware approaches to milk-based nutritional support in both term and preterm neonates.

In this study, we performed a paired two-stage quantitative proteomic analysis of milk samples collected within the first 10 days postpartum and during the fifth week of lactation in mothers of preterm and term infants. The aim was to characterize stage-associated proteomic changes within each cohort and to assess overlap and divergence at both protein and pathway levels.

## 2. Materials and Methods

### 2.1. Study Population and Study Design

The study included 40 lactating mothers whose infants were hospitalized in the Neonatal Intensive Care Unit (NICU), Department of Pediatrics, Jagiellonian University Medical College, Kraków, Poland, between October 2020 and November 2021. Eligible mothers were recruited consecutively among women whose infants were admitted to the NICU during the study period. Potential participants were screened according to the predefined inclusion and exclusion criteria and were approached by a member of the research team during their infant’s hospitalization. The study objectives and procedures were explained to all eligible mothers, and written informed consent was obtained prior to enrollment. Two groups were enrolled: mothers of very preterm infants born at <32 weeks’ gestation (preterm group) and mothers of term infants born between 37 and 42 weeks’ gestation (term group). The mean standard deviation of the protein abundance measurements, normalized to the mean intensity of the MS signal, was estimated at 0.5 based on previous measurements. Under this assumed variance, a statistical power of 0.8 is achieved for a two-sided *t*-test with *n* = 16 biological replicates per experimental group (quantitative significance cut-off at absolute fold change of 1.5). Thus, the presented study was conducted with a margin of *n* = 20 participants per group.

Inclusion criteria were as follows: (i) delivery at <32 weeks’ gestation (preterm group) or 37–42 weeks’ gestation (term group); (ii) declared intention to breastfeed; and (iii) admission of the infant to the neonatal unit within the first 10 days of life.

Exclusion criteria were as follows: (i) gestational diabetes or pre-existing diabetes; (ii) phenylketonuria in the infant; and (iii) other severe maternal conditions potentially affecting lactation, as determined by the investigator (e.g., severe postpartum hemorrhage, severe preeclampsia/eclampsia, sepsis or severe perinatal infection, and active oncological disease requiring treatment).

Each participating mother provided two paired milk samples collected at predefined time points: within the first 10 days postpartum (early lactation) and during the fifth week postpartum (established lactation).

The study was conducted in accordance with the Declaration of Helsinki. The study protocol was approved by the Ethical Committee of the Jagiellonian University Medical College (approval No. KBET/1072.6120.196.2020, 25 June 2020). Written informed consent was obtained from all participating women prior to enrollment.

The sample size was determined in the original study protocol designed to compare protein abundance between preterm and term milk. For the present paired longitudinal analysis, all available complete paired samples (*n* = 20 per cohort) were included. The present analysis represents a secondary longitudinal evaluation of an existing cohort originally powered for between-group comparisons. Although no separate power calculation was performed for within-cohort temporal analyses, all available complete paired samples were included to maximize statistical robustness of the within-subject comparisons.

### 2.2. Sample Preparation

#### 2.2.1. Milk Sample Collection

All milk samples were collected at noon following a minimum fasting period of four hours to reduce short-term dietary variability, although proteomic composition is not expected to exhibit major acute postprandial fluctuations. Milk expression was performed in the Neonatal Intensive Care Unit, where the infant was hospitalized. Each mother was provided with a sterile, single-use milk collection set. Milk was expressed using an electric breast pump (Medela Symphony, Medela AG, Baar, Switzerland) with disposable Symphony one-day pump sets for a duration of 10 min. From each expressed sample, 5 mL of milk was collected and immediately stored at −80 °C until further analysis. The same collection protocol was applied identically at both sampling time points.

#### 2.2.2. Milk Serum Separation

To minimize interference from milk fat globules and casein micelles during proteomic analysis, milk serum was isolated prior to protein extraction. Frozen samples were thawed at room temperature and centrifuged at 1500× *g* for 10 min to remove the upper lipid layer. The resulting supernatant was carefully collected and subjected to ultracentrifugation at 100,000× *g* for 90 min at 4 °C using a Beckman L-60 ultracentrifuge (Beckman Coulter, Brea, CA, USA). The clarified milk serum fraction was collected and used for subsequent proteomic analysis. The serum separation procedure was performed identically for samples from both lactation time points.

#### 2.2.3. Mass Spectrometry and Protein Quantification

Quantitative proteomic measurements were performed according to the protocol described previously [18]. Briefly, milk samples (20 μL) were mixed with 80 µL of lysis buffer (2.5% SDS, 60 mM DTT in 0.1 M Tris-HCl pH 7.6), vortexed, incubated at 95 °C for 5 min and clarified by centrifugation at 14,000× *g* for 30 min. Next, a volume containing 70 µg of total protein was transferred to Microcon-30 kDa centrifugal filter units (Merck, Darmstadt, Germany), denaturated with 8 M urea in 0.1 M Tris-HCl pH 8.5 and digested to peptides with a use of filter-aided sample preparation (FASP) protocol [19] using trypsin and endoproteinase Lys-C as proteolytic enzymes. Aliquots containing equal amounts of total peptides were desalted on 96-Well MiniSpin C18 columns (Harvard Apparatus, Holliston, MA, USA). Peptides (1 µg) were injected onto a nanoEase M/Z Peptide BEH C18 75 µm i.d. × 25 cm column (Waters, Milford, MA, USA) via a trap column nanoEase M/Z Symmetry C18 180 µm i.d. × 2 cm column (Waters) and separated by reversed-phase nano-chromatography by linear acetonitrile gradient and applied to a TripleTOF 6600+ (Sciex, Framingham, MA, USA) mass spectrometer operating in data-independent acquisition (DIA) mode. Spectra were collected in full scan mode (400–1250 Da), followed by one hundred SWATH MS/MS scans using a variable precursor isolation window approach, with *m*/*z* windows ranging from 6 to 90 Da. Data were filtered by 1% FDR on peptide and protein level, while quantitation and interference correction were done on the MS2 level in Spectronaut 20 [20]. Protein grouping was performed based on the ID picker algorithm [21].

### 2.3. Statistical Analysis of the Clinical Data

Categorical variables are presented as numbers and percentages. Continuous variables are expressed as mean and standard deviation (SD) or as median with first and third quartiles (Q1–Q3), as appropriate. Normality of distribution was assessed using the Shapiro–Wilk test.

Differences between groups were analyzed using Student’s *t*-test for normally distributed variables and the Mann–Whitney U test for non-normally distributed variables. JMP^®^, Version 17.1.0 (JMP Statistical Discovery, SAS Institute Inc., Cary, NC, USA) was used for all statistical analyses.

### 2.4. Mass Spectrometric Raw Data Analysis, Spectral Library Generation and SWATH Quantitation

Only proteins that were identified in at least 80% of the collected samples by at least two unique peptides were selected for further quantitative proteomic analysis to create a robust and reliable quantitative dataset. For the abovementioned set of proteins we performed a missing value imputation using a global imputation strategy where the missing values were imputed based on a random sampling from a distribution of low abundant signals taken throughout the entire experiment. For quantitative proteomics, two-sample *t*-tests were performed with false discovery rate (FDR) correction using the Storey method in Spectronaut [22]. Proteins with q < 0.05 were considered statistically significant and an absolute fold change value greater than 1.5, as reported in the corresponding result tables. Functional grouping and pathway annotations were performed using ClueGO [23] under the Cytoscape 3.8.2 software environment [24] with the use of PINE software 2.3.0 [25]. Human diseases (release 17 February 2020), CORUM-3.0 (release 3 September 2018), KEGG (release 17 February 2020), REACTOME (release 17 February 2020) and WikiPathways (release 17 February 2020) ontologies/pathways were used in the analysis. The enrichment results were validated by enrichment/depletion two-sided geometric statistical test with Bonferroni step down as *p*-value correction method. Minimum and maximum GO levels were set at 3 and 8, respectively, with the cluster criteria of a minimum of 3 genes constituting a minimum of 4% of the GO term. The Kappa score threshold was set as 0.4.

## 3. Results

### 3.1. Study Population and Maternal Characteristics

Baseline maternal, pregnancy, and neonatal characteristics are summarized in Table 1.

No significant differences were observed between mothers of preterm and term infants with respect to gravidity, parity, maternal age, educational level, smoking status, or previous breastfeeding history. As expected, maternal gestational weight gain was significantly lower in the preterm group (*p* = 0.0003).

Consistent with the study design, infants’ gestational age and birth weight differed significantly between groups (both *p* < 0.0001). Mode of delivery also differed between cohorts, with cesarean section more common in the preterm group (*p* = 0.0084).

Given the limited sample size, no multivariable adjustment for potential confounders was performed.

### 3.2. Overview of Proteomic Profiling and Stage-Associated Differential Abundance

**Figure 1:**
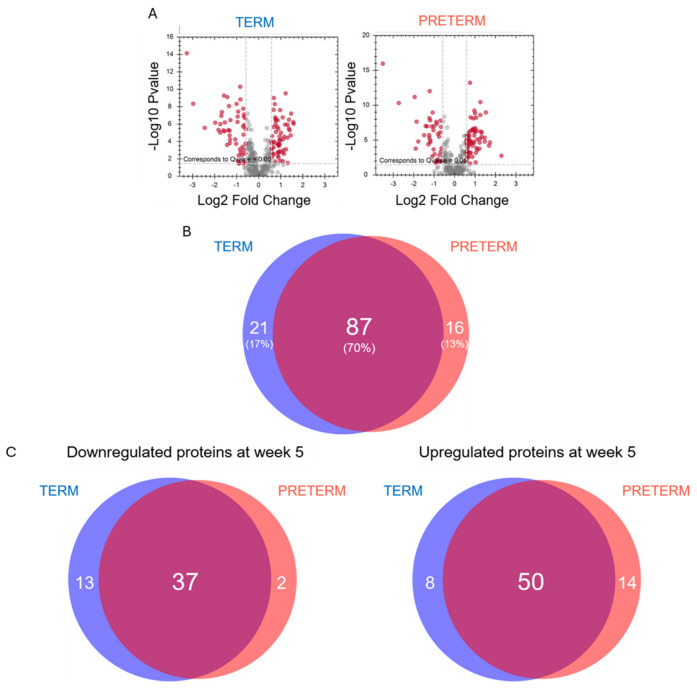
Details of quantitative proteomic measurements in human milk. Volcano plots summarize the magnitude and statistical significance of longitudinal protein abundance changes between early lactation and week 5 in term and preterm milk serum samples. (**A**). The Venn diagram illustrates overlap between cohorts among all significantly altered proteins, presented as absolute numbers and percentages, demonstrating extensive shared longitudinal proteomic remodeling (red—statistically sinificant proteins, grey—non-statistically significant proteins) (**B**). Separate Venn diagrams for decreased and increased proteins further demonstrate substantial inter-cohort overlap and concordant directionality of longitudinal changes across lactation (**C**).

Proteomic profiling generated quantitative data for a broad panel of milk serum proteins across all analyzed samples. The post hoc evaluation of the experimental data showed that the normalized standard deviation observed between the experimental groups ranged from 0.503 to 0.563. Therefore, the achieved statistical power to detect differences in measured protein levels was 0.82 in the term-born neonate group and 0.84 in the preterm infant group. After application of the predefined statistical and fold-change criteria, 108 proteins met the threshold for stage-associated differential abundance in the term cohort, including 58 proteins with increased abundance and 50 proteins with decreased abundance at week 5. In the preterm cohort, 103 proteins met these criteria, including 64 proteins with increased abundance and 39 proteins with decreased abundance. Full lists of differentially abundant proteins in the term and preterm cohorts are provided in Supplementary Table S1. Thus, both groups showed substantial and broadly comparable degrees of longitudinal proteomic remodeling across lactation. We next examined to what extent these changes reflected a shared temporal program and to what extent they were cohort-specific. Visual inspection of the overlap analysis suggested that most longitudinal proteomic changes were shared between cohorts, prompting a more detailed assessment of common and cohort-specific remodeling patterns (Figure 1).

### 3.3. Overlap of Stage-Associated Proteomic Remodeling Between Term and Preterm Milk

Comparison of the two longitudinal protein sets showed that most stage-associated changes were shared between cohorts. Of the proteins meeting differential-abundance criteria, 87 were shared between cohorts, corresponding to 80.6% of the term set and 84.5% of the preterm set. In contrast, 21 proteins were unique to the term cohort and 16 to the preterm cohort. Notably, all shared proteins showed concordant directionality of change across lactation in both groups. These findings indicate that stage-associated remodeling of the human milk serum proteome is dominated by a common trajectory, with only limited cohort-specific divergence.

### 3.4. Pathway-Level Interpretation of Shared and Cohort-Specific Remodeling

#### 3.4.1. Shared Pathways Associated with Proteins Increased at Week 5

**Figure 2:**
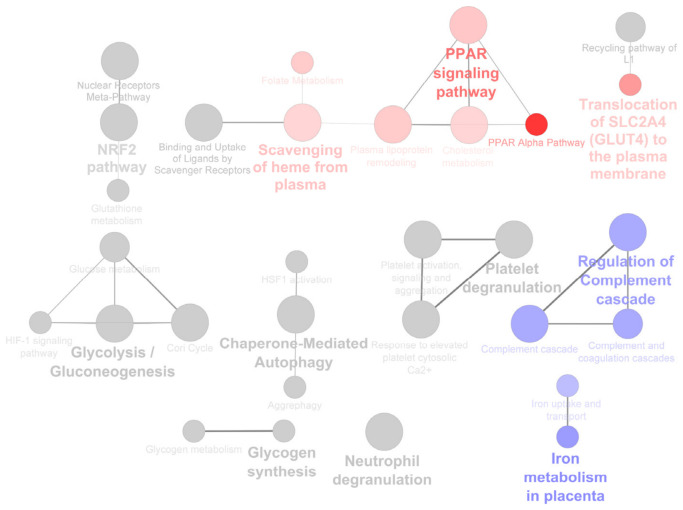
Functional enrichment network of processes identified by regulated milk proteins. Functional analysis was performed using proteins regulated in both groups as separate clusters. Among the identified signaling pathways, the majority did not exhibit specificity for either the term or preterm group (gray), which was associated with the high concordance of temporal changes in protein composition observed in both groups. The term group (blue) showed biased specificity towards the regulation of the complement cascade and iron turnover, whereas in the preterm group (red), PPAR activity-dependent metabolic processes and heme uptake were specifically regulated. Functional specificity bias was assigned based on the number of proteins enriched in a given process for each experimental group. Highlighted (bold) pathways represent the leading term in specific sub-category. Abbreviations: PPAR, peroxisome proliferator-activated receptor; NRF2, nuclear factor erythroid 2-related factor 2; SLC2A4 (GLUT4), solute carrier family 2 member 4 (glucose transporter type 4).

Among the proteins that increased at week 5, enrichment analysis identified a common set of significantly enriched biological processes in both the term and preterm cohorts. The shared enriched terms comprised hydrogen peroxide catabolic process, response to oxidative stress, cellular oxidant detoxification, and glycolytic process (Figure 2).

Representative proteins driving these shared pathway enrichments are presented in Table 2 (term) and Table 3 (preterm).

Notable shared upregulated proteins included FABP3, IDH1, FASN, and NAMPT, which showed approximately 2.5–2.9-fold upregulation across both cohorts. The antioxidant enzyme PRDX5 and glycolytic enzymes PGD, LDHB, and TKT were similarly increased, supporting coordinated metabolic adaptation. In the preterm cohort, S100A8 (4.90-fold) and LYZ (3.32-fold) were among the most prominently upregulated proteins.

#### 3.4.2. Shared Pathways Associated with Proteins Decreased at Week 5

Among proteins decreased at week 5, enrichment analysis identified a common set of significantly enriched biological processes in both the term and preterm cohorts. The shared enriched terms included innate immune response in mucosa, antibacterial humoral response, antimicrobial humoral immune response mediated by antimicrobial peptide, defense response to Gram-positive bacterium, modification-dependent protein catabolic process, and nucleosome assembly (Figure 2).

Proteins driving shared downregulated pathway enrichments are detailed in Table 4 (term) and Table 5 (preterm).

The most prominently decreased proteins in both cohorts included extracellular matrix components (TNC: 9.61-fold term, 11.41-fold preterm), membrane glycoproteins (CD63: 7.86-fold term, 6.63-fold preterm), and mucosal proteins (MUC1, MUC4), consistent with relative reduction in tissue remodeling and mucosal defense processes during the transition to established lactation.

### 3.5. Cohort-Specific Pathway Signals

**Figure 3:**
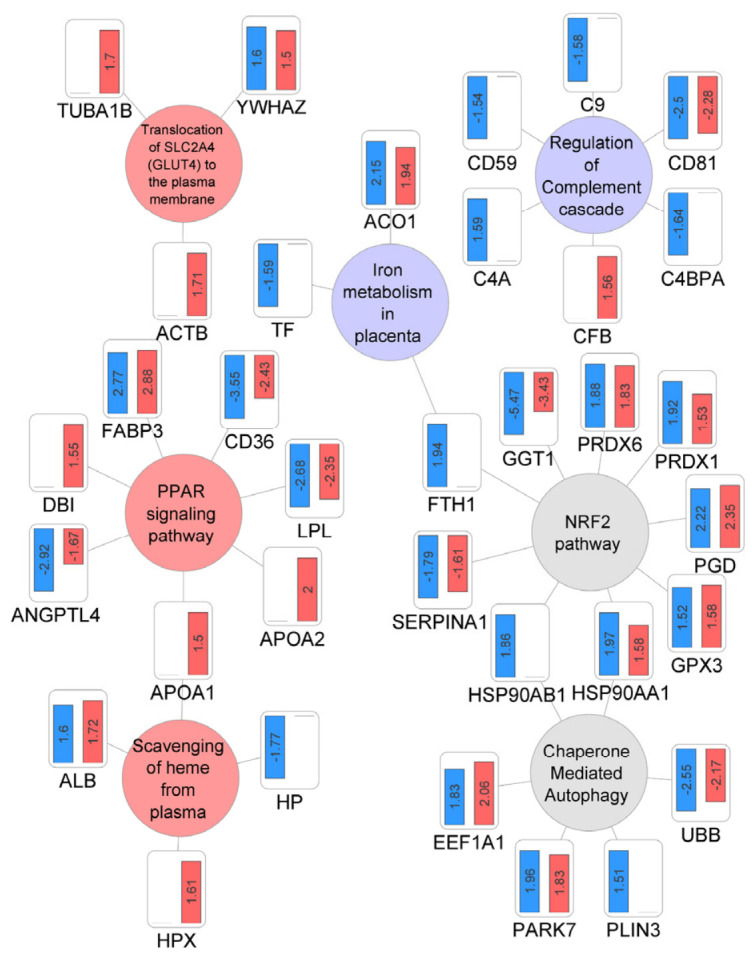
Quantitative details of regulated processes derived form the milk proteome. Bar charts collect the estimated statistically significant fold changes of proteins during the observation period in term (blue) and preterm (red) groups, respectively. Abbreviations: PPAR, peroxisome proliferator-activated receptor; NRF2, nuclear factor erythroid 2-related factor 2; SLC2A4 (GLUT4), solute carrier family 2 member 4 (glucose transporter type 4).

Despite the extensive overlap in stage-associated protein changes, selected pathway-level signals differed between cohorts. The NRF2 pathway, chaperone-mediated autophagy, and iron metabolism in placenta showed a predominant increase in the term cohort, whereas regulation of complement cascade showed a predominant decrease in the term cohort (Figure 3). In contrast, scavenging of heme from plasma and translocation of SLC2A4 (GLUT4) to the plasma membrane showed a predominant increase in the preterm cohort. The PPAR signaling pathway was represented in both cohorts without a clear cohort-restricted directional predominance. These cohort-selective pathway-level signals are summarized in Table 6.

## 4. Discussion

### 4.1. Main Findings and Interpretation

Our findings indicate that stage-associated remodeling of the human milk serum proteome is predominantly driven by a shared temporal program across both cohorts. Most changes occurred in the same direction, with approximately 80% of differentially abundant proteins shared between groups and only limited cohort-specific divergence at the pathway level. This pattern is consistent with the broader view of human milk as a dynamic biological system whose composition evolves across lactation in response to changing developmental and functional demands [9,10]. Rather than supporting distinct proteomic trajectories in term and preterm milk, the data suggest that longitudinal maturation is the primary organizing framework. Gestational-age-related differences appear to reflect more selective modulation, consistent with previous proteomic studies demonstrating complex interactions among genetic, environmental, and temporal factors [26]. These findings highlight the importance of structured longitudinal designs, as biologically meaningful signals may be obscured when lactation stage is treated as a secondary rather than a central analytical variable [27]. However, formal testing of time-by-group interactions was not performed, and therefore differences in the magnitude of longitudinal change between cohorts cannot be excluded.

Beyond characterizing compositional change, the milk proteome encodes a broad repertoire of biological functions that are directly relevant to infant development. Milk proteins participate in nutrient transport and bioavailability through multiple mechanisms: lactoferrin facilitates iron acquisition by binding and delivering iron to specific intestinal receptors, α-lactalbumin acts as a zinc-binding protein supporting mineral absorption, and osteopontin promotes intestinal maturation and modulates cytokine responses [28,29]. Growth-promoting effects are mediated by trophic factors including EGF and IGF-1, which drive intestinal epithelial proliferation and systemic anabolism, while human milk feeding has been shown to promote fat-free mass deposition in very-low-birth-weight preterm infants compared with formula feeding, with implications for long-term metabolic and neurodevelopmental outcomes [30,31]. Neurodevelopmental benefits associated with human milk feeding have been linked, at least in part, to long-chain polyunsaturated fatty acids, bioactive peptides, and protein-associated signaling molecules that support myelination, synaptic development, and the establishment of the microbiota–gut–brain axis [32]. Immune protection is conferred principally by secretory IgA, lactoferrin, and lysozyme, which together provide passive mucosal immunity and antimicrobial defense during the period of immunological immaturity in early infancy [28]. The observed stage-associated remodeling may represent an adaptation of milk composition to the infant’s developmental needs. Antimicrobial and structural proteins were enriched early in lactation, whereas metabolic and redox-related proteins increased by week 5.

### 4.2. Methodological Considerations

A major methodological strength of the present study lies in its paired within-subject design, in which each mother contributed two samples obtained at predefined stages of lactation and each sample was analyzed individually rather than pooled. This design enabled intra-individual proteomic trajectory analysis whilst preserving biological heterogeneity that pooled approaches would obscure. This is particularly relevant in human milk research, where interpretation depends not only on average compositional changes but also on recognizing human milk as a dynamic biological system shaped by both temporal change and interindividual variability, which has been shown to be substantial in previous proteomic studies [12,27]. Analyzing individual paired samples therefore provides a more direct view of longitudinal proteomic remodeling and reduces the risk that observed differences reflect averaging effects or static between-group contrasts. This distinction is critical: whereas our previous work compared preterm and term milk cross-sectionally at each stage [18], the present study isolates within-cohort temporal dynamics. The use of quantitative DIA proteomics further strengthened this framework by enabling consistent protein quantification across samples while preserving individual-level information central to the study question [20]. Importantly, unlike previous studies based on pooled or cross-sectional designs, the present analysis captures within-subject longitudinal dynamics in parallel term and preterm cohorts.

### 4.3. Shared Longitudinal Remodeling

The predominance of a shared temporal program was the most salient biological signal in our data. A total of 108 proteins in the term cohort and 103 in the preterm cohort met criteria for stage-associated differential abundance, with approximately 80% overlap and largely concordant directionality of change across lactation. This pattern indicates that lactation stage is a major determinant of proteomic remodeling in human milk serum, whereas gestational category exerts a more selective modulatory effect.

This interpretation is consistent with the previous literature showing that lactation is associated with systematic compositional change in human milk proteins. For example, immunoglobulins such as sIgA, IgM, and IgG decline during early lactation, alongside reductions in α-lactalbumin, lactoferrin, and β-casein, while lysozyme increases [11]. Proteomic studies have further demonstrated stage-dependent shifts in milk composition [13], together with substantial interindividual variability [12].

However, most previous studies have relied on pooled samples or cross-sectional designs and have not examined longitudinal changes at the individual level in parallel term and preterm cohorts. While large-scale proteomic analyses have confirmed extensive remodeling across lactation and identified broad functional transitions from immune-related to metabolic pathways [13,33], they provide limited insight into within-subject temporal dynamics.

Against this background, our paired, non-pooled analysis demonstrates that the dominant biological signal is not between-group divergence, but shared longitudinal remodeling across lactation in both cohorts.

### 4.4. Pathway-Level Insights

Shared pathways that increased at week 5 in both cohorts were primarily related to redox homeostasis and metabolic adaptation, including oxidative stress response, cellular detoxification, and glycolysis. This indicates that the observed temporal remodeling extends beyond individual proteins and reflects coordinated biological processes associated with the transition from early to established lactation. Similar functional shifts have been reported in previous proteomic studies, with later milk showing greater representation of biosynthetic and metabolic pathways [33,34]. These findings are consistent with evidence indicating that longitudinal change is a major driver of variation in the human milk proteome [26]. This pattern may reflect increasing metabolic demands of the mammary gland associated with sustained milk production and biosynthetic activity rather than cohort-specific effects.

Shared pathways that were less represented at week 5 in both cohorts were mainly related to mucosal and antimicrobial defense, including innate immune response and antibacterial humoral activity. This supports a relative shift from a defense-oriented early milk profile towards a more stabilized functional composition, rather than a loss of immune activity per se. This interpretation is consistent with evidence that early lactation is characterized by strong mucosal and antimicrobial protection [35,36,37], whereas later milk reflects a more mature secretory profile, with a transition towards metabolic and regulatory function [14]. Accordingly, the relative reduction in mucosal and antimicrobial pathways may correspond to the transition from colostrum-driven immune protection towards a more stable nutritional and regulatory profile.

### 4.5. Cohort-Specific Signals

Cohort-specific pathway signals were evident but modest in scope relative to the extensive shared remodeling observed across lactation. Within the term cohort, the predominant changes included increased representation of the NRF2 pathway, chaperone-mediated autophagy, and iron metabolism in placenta, together with a more marked decrease in regulation of complement cascade. By contrast, the preterm cohort showed a more prominent increase in the scavenging of heme from plasma and translocation of SLC2A4 (GLUT4) to the plasma membrane, whereas the PPAR signaling pathway was represented in both cohorts without a clearly cohort-restricted directional pattern. Taken together, these findings suggest that cohort-specific signals reflect selective modulation of particular biological processes rather than a globally distinct proteomic trajectory.

These findings indicate that gestational-age-related differences operate primarily at the level of selective pathway modulation and are superimposed on a broadly shared temporal program of proteomic maturation. This is consistent with previous studies showing that differences between term and preterm milk are strongly influenced by sampling stage and analytical design [15,34,38]. When interpreted alongside our previous stage-specific analysis, the present results further suggest that, once longitudinal within-cohort remodeling is explicitly modeled, cohort-specific differences are better understood as secondary modifiers of a common maturational trajectory rather than evidence of fundamentally distinct proteomic programs [18].

Previous studies have identified differences between preterm and term milk, but these have frequently been derived from pooled samples or cross-sectional comparisons at predefined lactation stages rather than from paired, individually analyzed longitudinal designs [13,15,34]. In this context, our findings indicate that, once within-subject temporal change is accounted for, the dominant biological signal is not broad divergence between cohorts but a largely shared program of proteomic remodeling.

This aligns with the concept of human milk as a dynamic biological system shaped by lactation stage, maternal–infant context, and study design rather than by gestational category alone [27]. Read together with our previous study, the present analysis addresses how proteomic remodeling unfolds within each cohort across lactation [18]. Although these data do not support immediate clinical extrapolation, they strengthen the case for a more individualized and temporally informed characterization of human milk biology, in line with current perspectives on preterm milk research [39].

### 4.6. Implications for Research and Practice

These findings highlight the importance of treating lactation stage as a central analytical variable in studies of human milk composition rather than as a secondary descriptor. Research on preterm milk is unlikely to be fully informative if based solely on group comparisons without accounting for temporal changes across lactation, particularly given that both protein composition and proteolytic activity vary with postnatal stage [27,38]. This has implications for study design, suggesting that comparisons between term and preterm milk should be stratified or modeled by lactation stage rather than treated as static group differences.

From a methodological perspective, this supports the development of study frameworks that better integrate temporal dynamics into the characterization of human milk, in line with current efforts to advance a more biologically informed understanding of preterm milk [39].

The present findings suggest that milk fortification protocols relying on compositional averages that do not account for lactation stage may incompletely reflect the biological profile of milk actually delivered to the preterm infant. A stage-aware characterization of human milk—integrating temporal proteomic dynamics alongside macronutrient content—may serve as a foundation for more individualized nutritional strategies in neonatal care.

### 4.7. Strengths

The principal strengths of this study include its paired within-subject design, the use of predefined sampling windows, and the separate evaluation of term and preterm cohorts within the same analytical framework. Each paired sample was analyzed individually rather than as pooled material, thereby preserving interindividual biological variability and allowing temporal remodeling to be assessed within each mother across lactation. Further strengths include the standardized approach to sample collection and processing, as well as the integration of pathway-level interpretation alongside protein-level findings, which enabled the results to be considered not only in terms of individual abundance changes but also as coordinated biological processes.

### 4.8. Limitations

Several limitations should be acknowledged. The study was conducted in a relatively small sample from a single center, and only two lactation time points were analyzed, which constrains the resolution with which temporal proteomic trajectories can be described. In addition, the analysis did not include multivariable adjustment or formal time-by-group interaction modeling, and therefore was not designed to determine whether the magnitude of longitudinal change differed significantly between cohorts after accounting for potential covariates. This limits the ability to formally assess whether observed similarities or differences between cohorts reflect true biological convergence or are influenced by statistical power. The proteomic findings were also not linked directly to infant clinical outcomes, and the pathway analysis remains inferential rather than mechanistic. Finally, the present work focused on the milk serum fraction rather than a whole-milk systems biology approach and thus does not capture the full complexity of other milk compartments or multimodal interactions.

## 5. Conclusions

Taken together, our findings indicate that the human milk serum proteome undergoes substantial longitudinal remodeling in both term and preterm lactation, with most changes following a shared temporal pattern. Although cohort-specific differences were detectable, these were comparatively limited and appeared to operate at a selective rather than global level. Within the constraints of the study design, these data support the interpretation that the transition from early to established lactation is governed predominantly by a common program of proteomic adaptation, with prematurity-related divergence superimposed upon this shared trajectory rather than representing a fundamentally distinct pattern of remodeling. The identified longitudinal proteomic patterns may help inform future studies exploring milk-derived biomarkers and personalized nutritional approaches for preterm and term infants.

## Figures and Tables

**Table 1:** Maternal, pregnancy, and neonatal characteristics of the study population.

	Mothers of Full-Term Infants (*n* = 20)	Mothers of Preterm Infants (*n* = 20)	*p*-Value
**Characteristics of Mothers**			
Parity; Me (Q1–Q3)	1 (1–2)	2 (1–2)	0.2057 ^M^
Maternal age [years]; mean (SD)	30.2 (5.5)	29.4 (6.1)	0.7346 ^T^
Maternal weight gain during pregnancy [kilograms]; Me (Q1–Q3)	15.0 (12.6–17.0)	9.5 (7.3–11.8)	0.0003 ^M^
Level of education; *n* (%)			
elementary	2 (10%)	1 (5%)	0.2356 ^F^
secondary	9 (45%)	14 (70%)	
university	9 (45%)	4 (20%)	
vocational	0 (0%)	1 (5%)	
Previously breastfeeding (whole group; *n* (%)	6 (30%)	10 (50%)	0.1967 ^P^
Breastfeeding a previous child (only if there was a previous child); *n* (%)	6 (86%)	10 (91%)	0.7324 ^P^
Interval between end of previous lactation [months]; mean (SD)	74 (45)	52 (32)	0.3299 ^T^
**Characteristics of the current pregnancy**			
Gestational age [weeks]; Me (Q1–Q3)	39 (38–40)	30 (29–31)	<0.0001 ^M^
Birth weight [grams], Me (Q1–Q3)	3020 (2843–3515)	1400 (1225–1575)	<0.0001 ^M^
Mode of delivery; *n* (%)			
Cesarean section	11 (55%)	18 (90%)	
Vaginal	9 (45%)	1 (5%)	0.0084 ^F^
Vaginal + cesarean section (twin pregnancy)	0 (0%)	1 (5%)	
Smoking; *n* (%)	1 (5%)	2 (10%)	0.5483 ^P^

Data are presented as mean ± SD or median (Q1–Q3), as appropriate, or as number (percentage). Between-group comparisons were performed using Student’s *t*-test (^T^) or the Mann–Whitney U test (^M^) for continuous variables and Pearson’s chi-squared test (^P^) or Fisher’s exact test (^F^) for categorical variables, as appropriate. Normality was assessed using the Shapiro–Wilk test.

**Table 2:** Top 15 proteins increased at week 5 in term milk.

UniProt Accession	Genes	Protein Descriptions	Q-Value	Fold Change
P00491	*PNP*	Purine nucleoside phosphorylase	6.38 × 10^−6^	2.96
P05413	*FABP3*	Fatty acid-binding protein, heart	1.18 × 10^−6^	2.77
O75874	*IDH1*	Isocitrate dehydrogenase [NADP] cytoplasmic	0.0001	2.73
P49327	*FASN*	Fatty acid synthase	3.50 × 10^−5^	2.64
Q16555	*DPYSL2*	Dihydropyrimidinase-related protein 2	3.95 × 10^−6^	2.59
P43490	*NAMPT*	Nicotinamide phosphoribosyltransferase	2.52 × 10^−5^	2.58
P30044	*PRDX5*	Peroxiredoxin-5, mitochondrial	0.044	2.49
Q96KP4	*CNDP2*	Cytosolic non-specific dipeptidase	1.09 × 10^−5^	2.48
P31949	*S100A11*	Protein S100-A11	0.0002	2.46
P49189	*ALDH9A1*	4-trimethylaminobutyraldehyde dehydrogenase	0.002	2.36
P30086	*PEBP1*	Phosphatidylethanolamine-binding protein 1	2.56 × 10^−8^	2.33
P07195	*LDHB*	L-lactate dehydrogenase B chain	1.06 × 10^−5^	2.30
P14550	*AKR1A1*	Aldo-keto reductase family 1 member A1	0.0001	2.23
P52209	*PGD*	6-phosphogluconate dehydrogenase, decarboxylating	0.006	2.22
P29401	*TKT*	Transketolase	0.003	2.16

Total differentially abundant proteins in term milk: 108 (58 increased, 50 decreased at week 5). Full list available in Supplementary Table S1.

**Table 3:** Top 15 proteins increased at week 5 in preterm milk.

UniProt Accession	Genes	Protein Descriptions	Q-Value	Fold Change
P24821	*TNC*	Tenascin	4.04 × 10^−12^	9.61
P08962	*CD63*	CD63 antigen	1.76 × 10^−7^	7.86
P19440	*GGT1*	Glutathione hydrolase 1 proenzyme	2.15 × 10^−5^	5.47
Q9NZH0	*GPRC5B*	G-protein coupled receptor family C group 5 member B	7.09 × 10^−6^	3.92
P16671	*CD36*	Platelet glycoprotein 4	3.27 × 10^−5^	3.55
P47710	*CSN1S1*	Alpha-S1-casein	5.76 × 10^−5^	3.35
O00560	*SDCBP*	Syntenin-1	9.57 × 10^−6^	3.34
Q08431	*MFGE8*	Lactadherin	1.86 × 10^−6^	3.24
P15941	*MUC1*	Mucin-1	8.55 × 10^−7^	3.18
P07996	*THBS1*	Thrombospondin-1	3.67 × 10^−5^	3.03
O15232	*MATN3*	Matrilin-3	4.02 × 10^−8^	2.99
Q9BY76	*ANGPTL4*	Angiopoietin-related protein 4	4.49 × 10^−5^	2.92
Q99102	*MUC4*	Mucin-4	6.79 × 10^−6^	2.90
P21926	*CD9*	CD9 antigen	0.0008	2.70
P06858	*LPL*	Lipoprotein lipase	4.70 × 10^−8^	2.68

Total differentially abundant proteins in preterm milk: 103 (64 increased, 39 decreased at week 5). Full list available in Supplementary Table S1.

**Table 4:** Top 15 proteins decreased at week 5 in term milk. Fold change values are presented as absolute ratios; directionality is indicated by the table heading.

UniProt Accession	Genes	Protein Descriptions	Q-Value	Fold Change
P05109	*S100A8*	Protein S100-A8	0.004	4.90
P61626	*LYZ*	Lysozyme C	0.0001	3.32
P05413	*FABP3*	Fatty acid-binding protein, heart	5.56 × 10^−8^	2.88
O75874	*IDH1*	Isocitrate dehydrogenase [NADP] cytoplasmic	8.86 × 10^−5^	2.79
P49327	*FASN*	Fatty acid synthase	8.35 × 10^−6^	2.50
P00491	*PNP*	Purine nucleoside phosphorylase	2.55 × 10^−5^	2.49
P43490	*NAMPT*	Nicotinamide phosphoribosyltransferase	6.50 × 10^−6^	2.46
P30086	*PEBP1*	Phosphatidylethanolamine-binding protein 1	4.03 × 10^−9^	2.40
P20061	*TCN1*	Transcobalamin-1	1.10 × 10^−7^	2.39
P31949	*S100A11*	Protein S100-A11	4.29 × 10^−5^	2.37
P52209	*PGD*	6-phosphogluconate dehydrogenase, decarboxylating	0.0001	2.35
P30044	*PRDX5*	Peroxiredoxin-5, mitochondrial	0.0006	2.32
P49189	*ALDH9A1*	4-trimethylaminobutyraldehyde dehydrogenase	0.0008	2.17
P07737	*PFN1*	Profilin-1	5.44 × 10^−6^	2.15
P29401	*TKT*	Transketolase	5.18 × 10^−5^	2.15

Total differentially abundant proteins in TTTotal differentially abundant proteins in term milk: 108 (58 increased, 50 decreased at week 5). Full list available in Supplementary Table S1.

**Table 5:** Top 15 proteins decreased at week 5 in preterm milk. Fold change values are presented as absolute ratios; directionality is indicated by the table heading.

UniProt Accession	Genes	Protein Descriptions	Q-Value	Fold Change
P24821	*TNC*	Tenascin	1.24 × 10^−13^	11.41
P08962	*CD63*	CD63 antigen	4.81 × 10^−9^	6.63
O15232	*MATN3*	Matrilin-3	8.12 × 10^−10^	3.88
P01903	*HLA-DRA*	HLA class II histocompatibility antigen, DR alpha chain	0.0006	3.74
Q9NZH0	*GPRC5B*	G-protein coupled receptor family C group 5 member	5.36 × 10^−7^	3.63
P19440	*GGT1*	Glutathione hydrolase 1 proenzyme	8.80 × 10^−5^	3.43
O00560	*SDCBP*	Syntenin-1	1.69 × 10^−5^	2.81
P07996	*THBS1*	Thrombospondin-1	1.68 × 10^−6^	2.67
P15941	*MUC1*	Mucin-1	1.84 × 10^−6^	2.55
P16671	*CD36*	Platelet glycoprotein 4	0.0002	2.43
P47710	*CSN1S1*	Alpha-S1-casein	1.64 × 10^−5^	2.37
Q08431	*MFGE8*	Lactadherin	3.22 × 10^−5^	2.36
P06858	*LPL*	Lipoprotein lipase	2.26 × 10^−7^	2.35
Q6WN34	*CHRDL2*	Chordin-like protein 2	2.07 × 10^−10^	2.33
P05090	*APOD*	Apolipoprotein D	4.13 × 10^−7^	2.29

Total differentially abundant proteins in preterm milk: 103 (64 increased, 39 decreased at week 5). Full list available in Supplementary Table S1.

**Table 6:** Summary of pathway-level directionality in term and preterm milk across lactation.

	Term	Preterm
PPAR signaling pathway	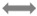	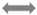
NRF2 pathway	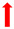	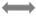
Regulation of complement cascade	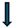	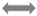
Scavenging of heme from plasma	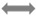	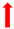
Chaperone-mediated autophagy	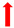	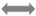
Iron metabolism in placenta	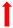	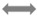
Translocation of SLC2A4 (GLUT4) to the plasma membrane	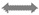	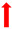

Abbreviations: PPAR, peroxisome proliferator-activated receptor; NRF2, nuclear factor erythroid 2-related factor 2; SLC2A4 (GLUT4), solute carrier family 2 member 4 (glucose transporter type 4). Red arrow—pathway showed increase in the cohort, blue arrow—pathway showed decrease in the cohort, grey arrow—pathway without a clear cohort-restricted directional predominance.

## Data Availability

The mass spectrometry proteomics data have been deposited to the ProteomeXchange Consortium via the PRIDE [40] partner repository with the dataset identifier PXD074649. Clinical and demographic data are not publicly available due to ethical and privacy restrictions.

## Supplementary Materials

The following supporting information can be downloaded at: https://www.mdpi.com/article/10.3390/nu18132199/s1, Table S1. Full list of differentially abundant proteins.
